# Delayed breast reconstruction with tram-flap and various modifications after radical mastectomy

**DOI:** 10.25122/jml-2021-0354

**Published:** 2021

**Authors:** Ivan Ivanovich Smolanka, Irina Yuriivna Bagmut, Michael Ivanovicha Sheremet, Andriy Oleksandrovich Lyashenko, Oleksii Volodimirovich Movchan, Ivan Ivanovich Smolanka, Anton Dmitrovich Loboda, Igor Leonidovich Kolisnyk, Larysa Petrivna Sydorchuk, Oleksandr Volodimirovich Lazaruk

**Affiliations:** 1. National Cancer Institute, Ministry of Health, Kyiv, Ukraine; 2. Kharkiv Medical Academy of Postgraduate Education, Kharkiv,Ukraine; 3. Surgery Department No.1, Bukovinian State Medical University, Chernivtsi, Ukraine; 4. Family Medicine Department, Bukovinian State Medical University, Chernivtsi, Ukraine; 5. Department of Pathology, Bukovinian State Medical University, Chernivtsi, Ukraine

**Keywords:** inflammatory breast cancer, intra-arterial chemotherapy, oncoplastic surgery, simultaneous breast plastic surgery

## Abstract

This study describes the experience of radical mastectomies with simultaneous breast reconstruction using TRAM flap in patients with inflammatory breast cancer. The study aimed to evaluate the effectiveness of primary breast reconstruction using the TRAM-flap procedure in patients with an inflammatory form of breast cancer. Our work is associated with some deviation from generally accepted standards: delayed breast reconstruction after radical mastectomy for inflammatory breast cancer. We describe the experience of radical mastectomies with the simultaneous reconstruction of the breast using a TRAM flap in patients with inflammatory breast cancer. This study included 12 patients diagnosed with breast cancer stages IIIB and IIIC. Almost all patients (eleven out of twelve patients) underwent radical mastectomy with one-stage reconstruction using a TRAM flap after chemotherapy. Two years later, one patient (8.3%) showed disease progression in the form of distant metastases in the bones of the spine. One patient (8.3%) had a regional relapse in the displaced flap near the postoperative scar. The rest of the patients (83.4%) showed no signs of continuing the disease. Patients with one-stage breast reconstruction improved socially, and their subjective well-being was better than those who underwent radical mastectomy without reconstruction. Experience in performing one-stage reconstructions in the surgical treatment of patients with inflammatory breast cancer is a reason for restrained optimism regarding the possibility and feasibility of these operations.

## Introduction

Inflammatory breast cancer is an urgent problem of modern oncology, occupying a relatively significant share (about 5%) in the total structure of breast cancer, with a rapid course and an unfavorable prognosis [1, 2].

The clinical picture of this form of breast cancer resembles an inflammatory process. There are thickening and hyperemia of the skin, an increase in the size of the affected gland, a local increase in temperature, and diffuse thickening of the gland tissue. A specific symptom is the occurrence of edema, which affects more than a third of the breast. In addition, damage to regional lymphatic collectors is characteristic. It is believed that regional lymph nodes are affected in 95% of cases, and supraclavicular lymph nodes in 40% [3–5]. The aforementioned is customary to distinguish between diffuse (truly inflammatory) and nodular forms of inflammatory forms of breast cancer. The first is associated with edema without a solid tumor, and the second means the presence of a tumor node with edema of the surrounding tissues. If tumor regression is achieved (preferably complete), surgery is performed. Its volume is not yet a subject of discussion. It is generally accepted to perform a radical mastectomy. This is due to the high risk of retaining residual tumors in the form of tumor emboli in the lymph and blood vessels of the breast after neoadjuvant treatment. Reconstructive surgery is usually postponed at least for the period of completion of adjuvant therapy after a radical mastectomy. Only a few reports are devoted to simultaneous reconstructions in the surgical treatment of inflammatory breast cancer [6–12]. 

Diagnosis of inflammatory breast cancer is based on generally accepted clinical and instrumental studies and histological verification methods. It is mandatory to perform mammography, and if necessary, scintimammography. In addition, doppler scanning, computed tomography, magnetic resonance imaging, and positron emission tomography are used to clarify the diagnosis. Histological verification of the diagnosis involves a trephine biopsy followed by a microscopic examination of the biopsy. In the absence of a tumor node (a truly inflammatory form of breast cancer), a puncture biopsy of regional lymphatic collectors is performed, or samples of breast tissue in the area of edema are examined. Treatment begins with neoadjuvant chemotherapy, and only in the case of a response (positive dynamics on the part of the tumor – complete or partial regression) to chemotherapy, the possibility of surgical intervention is considered [13–18]. 

Treatment of inflammatory breast cancer according to the latest recommendations of the National Comprehensive Cancer Network (NCCN), American Society of Clinical Oncology (ASCO), European Society for Medical Oncology (ESMO), should be comprehensive using all the main methods of treatment [19]. 

It should be noted that the very possibility of a surgical method in the treatment of inflammatory breast cancer is still a matter of controversy since some experts are convinced that surgical interventions are inappropriate for this form of breast cancer. However, most experts think the surgical stage is necessary [19]. If neoadjuvant chemotherapy is ineffective, radiotherapy is prescribed, after which the feasibility of surgical intervention is reassessed [20].

According to our observations, surgical intervention is possible only after the disappearance of such clinical manifestations of widespread (>30%) edema of the breast skin, satellites on the skin of the breast, metastases in the parasternal, or supraclavicular lymph nodes, and edema of the upper limb.

One study involved 23 patients who underwent reconstruction after mastectomy, 14 patients who underwent surgery simultaneously, and 9 patients who delayed reconstruction [21]. The most common intervention was TRAM grafting with a TRAM flap (18 patients), three endoprosthesis, and two plastic surgeries with a thoracodorsal flap. The median survival rate was 44 months. There were no statistically significant deviations in disease-free and overall survival rates for both groups. It was noted that 90% of patients were satisfied with reconstruction results. At the same time, another study reported 6 out of 10 cases of local recurrences observed after one-stage reconstructions with a musculocutaneous flap after a year. No other negative consequences were observed [22].

In general, momentary reconstructions are treated with caution in the oncological community with this form of breast cancer [23]. However, we see that already a year after operations with one-stage reconstruction, there is a rather high percentage of local recurrences or metastatic disease [24]. This article describes breast reconstruction experience using simultaneous TRAM-flap procedures in the surgical treatment of patients with inflammatory breast cancer. The study aimed to evaluate the effectiveness of primary reconstructive breast plastic methods using TRAM-flap in patients with an inflammatory form of breast cancer.

## Material and Methods

Our work is associated with some deviation from generally accepted standards: delayed breast reconstruction after radical mastectomy for inflammatory breast cancer. We describe the experience of radical mastectomies with the simultaneous reconstruction of the breast using TRAM flap in patients with inflammatory breast cancer. During 2010–2018, 12 patients had surgery for breast cancer stages IIIB and IIIC using this technique. Of these, four patients were diagnosed with breast cancer T4bN1-2M0 (edema, which is less than a third of the breast, “transition” to the inflammatory form), six patients – T4dN1-2M0, one patient – T4dN3M0, and another patient – T4dN2M1. The attraction to this group of patients with distant foci was due to the young age of the women, purposeful motivation to preserve the gland, and the presence of only a single skeletal metastasis, which became sclerosed after treatment. Reconstructive interventions were performed only if these points were reached: the disappearance of clinical manifestations such as widespread edema of the breast skin (>30%), satellites on the skin of the breast, metastases in the parasternal or supraclavicular lymph nodes, edema of the upper limb, complete regression (CR) or partial regression (PR) with instrumental monitoring using mammography. If necessary, Doppler scanning, computed tomography, magnetic resonance imaging, positron emission tomography after preliminary neoadjuvant chemotherapy. All twelve patients had affected regional lymph nodes before treatment (Table 1); 11 patients met criteria N2 and one patient N3.

**Table 1.** Distribution of patients with inflammatory breast cancer depending on the lesion of regional lymphatic collectors.

Regional lymph nodes prevalence rates, N	Number of patients, n (%)	Standard deviation, p
**N0-1**	0	-
**N2**	11 (91.7±4.85)	>0.05
**N3**	1 (8.3±6.24)	>0.05
**All**	12 (100%)	-

At the first stage of treatment, all patients underwent neoadjuvant therapy: 8 patients’ intra-arterial chemotherapy and 4 patients’ systemic chemotherapy. Intra-arterial chemotherapy was performed after preliminary angiography and selective catheterization of the vessel, which predominantly feeds the tumor (Table 2).

**Table 2.** Catheterization of the vessels feeding the tumor in patients with inflammatory breast cancer.

**Vessel catheterization**	**Number of patients, n (%)**	**Standard deviation, p**
**A. thoracica lateralis**	1 (8.3±6.24)	>0.05
**A. thoraco-dorsalis**	2 (16,7±7.76)	>0.05
**A. thoracica interna**	3 (25.0±5.12)	>0.05
**Any 2 arteries simultaneously**	2 (16.7±7.76)	>0.05
**All**	8 (100%)	-

In one case, the thoracic lateralis vein was catheterized, in 2 more patients – the thoracodorsalis, and in 3 other cases – an internal thoracic arteria, in the other 2 cases, catheterization of two feeding arteries were performed simultaneously. The regimen included docetaxel and cyclophosphamide with 6 to 8 cycles and 21 days apart. Another 4 patients underwent systemic chemotherapy according to the DC scheme. The effect of the treatment was assessed according to the data of clinical and instrumental studies under the RECIST criteria after every two cycles of therapy, data from trephine biopsies from different parts of the affected gland.

**Figure 1.**
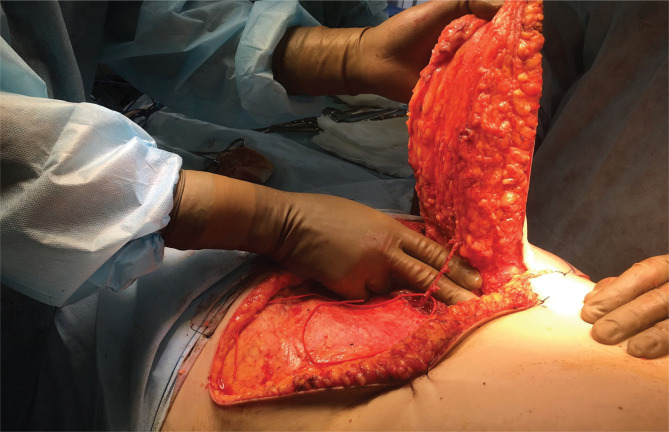
The vascular anastomosis was made between the inferior epigastric artery and a vein with the internal thoracic artery and vein on the left.

The lower epigastric vessels and their collaterals following intra-arterial chemotherapy were assessed by performing preoperative duplex ultrasound scanning with linear transducers with a frequency of 7.5, 10, and 12.5 MHz and the standard marking of the transverse abdominal flap. After intra-arterial and systemic chemotherapy, eleven out of twelve patients underwent radical mastectomy with simultaneous reconstruction using a TRAM flap. Given the prevalence of the process, a prerequisite was the study of the purity of the edges after performing a radical mastectomy. After performing a mastectomy in Madden’s modification, a TRAM flap was isolated on the contralateral leg, moved to the postoperative wound through the formed tunnel, and fixed. The defect of the rectus abdominis muscle was eliminated with a prolene mesh. Standard special treatment was prescribed in the postoperative period depending on the molecular genetic subtype. According to the scheme given above, all patients underwent adjuvant systemic chemotherapy at least four courses. Patients with hormone-dependent tumors started endocrine therapy with hypothalamic releasing factor analogs combined with antiestrogens or aromatase inhibitors. Patients with confirmed positive Her2/neu status were treated with trastuzumab in standard doses. One patient with a “transition” to an inflammatory form after systemic chemotherapy and after achieving complete regression underwent plastic surgery using a perforating TRAM flap (MS-TRAM). A perforating flap of the inferior epigastric artery was isolated, including the skin, subcutaneous tissue feeding the artery with an accompanying vein, perforating vessels, and surrounding muscle areas. The vascular anastomosis was made between the inferior epigastric artery and a vein with the internal thoracic artery and vein on the left. (Figure 1).

## Results

The age of women was in the range of 23–52 years. Generally speaking, all candidates for reconstructive interventions had a relatively young age and a strong motivation to preserve the gland at all costs. In 7 patients, the tumor was localized in the left breast, in 5 patients – in the right. The histological picture after performing trephine biopsy showed glandular ductal adenocarcinoma in 8 cases, lobular infiltrating carcinoma in 4 cases. Immunohistochemically, three times negative cancer was noted in 3 patients, subtype “HER2” – in 2, luminal B subtype – in 3, and luminal A subtype was detected in 4 patients (Table 3).

**Table 3.** Distribution of patients with swollen breast cancer in depending on receptor.

**Hormonal subtype of the tumor**	**Number of patients, n (%)**	**Standard deviation, p**
**Luminal A**	4 (33.3±6.15)	>0.05
**Luminal B**	3 (25.0±5.12)	>0.05
**Triple negative**	3 (25.0±5.12)	>0.05
**HER 2**	2 (16.7±7.76)	>0.05
**All**	12 (100%)	-

After neoadjuvant intra-arterial chemotherapy, complete regression was noted in 3 patients and partial regression in 5 cases. In patients with systemic administration of chemotherapy drugs, 2 patients showed complete regression, and 2 patients partial regression. It is noteworthy that with a truly inflammatory form of breast cancer, 2 cases of a complete response to neoadjuvant treatment were recorded. After performing the mastectomy, a “clean margin” of the resection (R0) was achieved in all cases. All surgical interventions were performed successfully, musculocutaneous flaps were viable, and a cosmetic effect was achieved that satisfied all patients. In the early postoperative period, there were two cases of marginal necrosis that required surgical correction. There was also a case of prolonged lymphorrhea in one patient. Three patients, at their request, underwent corrective plastic interventions (removal of postoperative scars and contralateral mastopexy), and five patients underwent a nipple-areola complex. Two years later, one patient (8.3%) showed disease progression in the form of distant metastases in the bones of the spine and small pelvis. One patient (8.3%) had a regional recurrence in the displaced flap near the postoperative scar. The rest of the patients (83.4%) showed no signs of continuing the disease.

## Discussion

When we are talking about postoperative complications after breast reconstruction with TRAM-flap, two cases of marginal necrosis required surgical correction, corresponding to the results of Susman *et al.* [25], and there was one case of prolonged lymphorrhea as suggested by Ercan Karacaoglu [26]. One patient (8.3%) showed disease progression in the form of distant metastases in the bones of the spine and small pelvis, and one patient (8.3%) had a regional recurrence in the displaced flap near the postoperative scar, which is within the results obtained by Zikiryakhodzhayev [27].

## Conclusions

The preliminary results of the study indicate the fundamental possibility of simultaneous breast reconstruction as a stage of surgical treatment in patients with inflammatory breast cancer. Such an approach in treating inflammatory forms of breast cancer is possible only when the effect is achieved (complete or partial tumor regression – according to instrumental monitoring methods), morphologically confirmed residuality, and disappearance of clinical manifestations of the inflammatory form of the disease after neoadjuvant treatment. Performing simultaneous reconstruction in patients of this group is a technically difficult moment. Risk in the surgical plan, compared to radical mastectomy, threatens a large number of postoperative complications.

However, as can be seen from the data, the number of complications and the results obtained are similar to those reported in separate sources. Patients with one-stage breast reconstruction improved socially; their subjective well-being was better than patients who underwent radical mastectomy without reconstruction. The obtained material does not claim to be exhaustive and is not a reason for categorical conclusions. Research data will be expanded and observations evaluated at a later date. The experience of performing one-stage reconstructions in the surgical treatment of patients with inflammatory breast cancer is a reason for restrained optimism about the possibility and expediency of these operations. At the same time, a limited number of observations, contradictory results of international reports force us to refrain from conclusions.

## Acknowledgements

### Conflict of interest

The authors declare that there is no conflict of interest.

### Ethical approval

The study was conducted according to the ethical principles of the Helsinki Declaration, GCP (Good Clinical Practice), and Law of Ukraine regarding medications approved by the Commission of Ethics from the National Cancer Institute (Minutes No. 7 of April 8, 2018).

### Consent to participate

Written informed consent was obtained for all participants in the study.

### Personal thanks

We want to thank professor Irina Yurievna Bagmut.

### Authorship

IVS contributed to work concept and design, data collection and analysis, writing the article, critical review, and final approval of the article. IYB contributed to work concept and design, critical review, and final article approval. MIS and AOL contributed to data collection and analysis, writing the article, and critical review. OVM contributed to work concept and design, data collection and analysis, writing the article, critical review, and final approval of the article. IVSJ contributed to data collection and analysis, writing the article. ADV, ILK, LPS, and OVL contributed to data collection and analysis, writing the article.
